# Mental and Reproductive Health Correlates of Academic Performance among Debre Berhan University Female Students, Ethiopia: The Case of Premenstrual Dysphoric Disorder

**DOI:** 10.1155/2017/9348159

**Published:** 2017-05-29

**Authors:** Sisay Mulugeta Alemu, Tesfa Dejenie Habtewold, Yohannes Gebreegziabhere Haile

**Affiliations:** ^1^Mental Health and Psychosocial Support Program, International Medical Corps, Dolo Ado, Ethiopia; ^2^Department of Epidemiology and Rob Giel Research Center, University of Groningen, Groningen, Netherlands; ^3^Department of Nursing, Debre Berhan University, Debre Berhan, Ethiopia

## Abstract

**Background:**

Globally 3 to 8% of reproductive age women are suffering from premenstrual dysphoric disorder (PMDD). Several mental and reproductive health-related factors cause low academic achievement during university education. However, limited data exist in Ethiopia. The aim of the study was to investigate mental and reproductive health correlates of academic performance.

**Methods:**

Institution based cross-sectional study was conducted with 667 Debre Berhan University female students from April to June 2015. Academic performance was the outcome variable. Mental and reproductive health characteristics were explanatory variables. Two-way analysis of variance (ANOVA) test of association was applied to examine group difference in academic performance.

**Result:**

Among 529 students who participated, 49.3% reported mild premenstrual syndrome (PMS), 36.9% reported moderate/severe PMS, and 13.8% fulfilled PMDD diagnostic criteria. The ANOVA test of association revealed that there was no significant difference in academic performance between students with different level of PMS experience (*F*-statistic = 0.08, *p* value = 0.93). Nevertheless, there was a significant difference in academic performance between students with different length of menses (*F*-statistic = 5.15, *p* value = 0.006).

**Conclusion:**

There was no significant association between PMS experience and academic performance, but on the other hand, the length of menses significantly associated with academic performance.

## 1. Introduction

Menstrual cycle is the orderly cyclic hormone production and parallel proliferation of the uterine lining to prepare for implantation of the embryo [1]. Most women report mood symptoms, behavioral changes, and physical symptoms during the menstrual cycle; however, these symptoms resolve for the rest of time between menstrual cycles. This group of symptoms are first named as premenstrual tension by Frank and later renamed to premenstrual syndrome by Greene and Dalton in 1953 [2].

There are more than 150 premenstrual symptoms registered in 10th revision of the International Statistical Classification of Diseases and Related Health Problems (ICD-10) [3]. Premenstrual syndrome (PMS) is the presence of at least one out of fourteen symptoms registered in the fifth text edition of Diagnostic and Statistical Manual of Mental Disorders (DSM-5) [4]. Premenstrual dysphoric disorder (PMDD), a severe form of PMS, is defined as the presence of at least five out of eleven DSM-5 mood or behavioral or physical symptoms with the substantial interference of daily activities [4].

Even though up to 80% of women experience mild to severe premenstrual symptoms [5–9], they consider these symptoms as a natural event [6] and do not seek medical advice [10]. According to the DSM-5 criteria, the prevalence of PMDD was 3 to 8% [11]. In addition, 18% of reproductive age women suffered from moderate to severe premenstrual symptoms [12].

Previous studies showed that biological, psychological, and social-cultural factors cause PMDD [13]. These factors include educational and marital status, genetic predisposition, age, pain during menses, amount of menstrual bleeding, history of physical and psychiatric illness, treatment seeking behavior, history of traumatic event, sleeping hour, physical exercise, and maternal history of PMS [14–26]. Women become symptomatic when the social and psychological stressors induce biochemical (hormone and neurotransmitter) changes [2].

Among others, school absenteeism and poor academic performance were the deleterious effects of PMDD [27]. Moreover, PMDD has been associated with suicide and accident rate, major depressive disorder, high job absenteeism, poor quality of life, and poor family and coworkers relationship [28, 29].

Despite a considerable effect on women daily functioning, the association between PMS experience and academic performance has given little attention [11]. Thus, the aim of this study was to investigate mental and reproductive health correlates of academic performance among female students.

## 2. Methods and Materials

### 2.1. Population and Procedure

Institution based cross-sectional study was conducted at Debre Berhan University from April to June 2015. Undergraduate students who enrolled in 2014/2015 full-time study, who were capable of independent communication, and who provided informed written consent were included. All students were selected by multistage cluster sampling technique (design effect = 2). First, 5 out of 8 colleges were selected by simple random sampling technique. Second, 14 departments representing 40% of departments at Debre Berhan University were selected using simple random sampling technique. Finally, student batches (1st, 2nd, and 3rd year) were selected randomly and data was collected from the entire students. The data was collected after the class.

### 2.2. Sample Size Determination

The sample size was determined by single population proportion formula considering the following assumptions: 27% prevalence of PMDD in Ethiopian university students [19] and 95% confidence level. After adjustment for the design effect of 2 and 10% nonresponse rate, the final sample size was 667.

### 2.3. Instrument

Data was collected from 14 departments using pretested self-administered questionnaire. The questionnaire has four parts: part 1, sociodemographic characteristics; part 2, menstrual cycle characteristics; part 3, treatment seeking behavior and general health status; part 4, premenstrual symptoms and functionality. Premenstrual symptoms screening tool (PSST) was used to assess PMDD. The PSST includes a list of premenstrual psychiatric and physical symptoms, and a measure of functional impairment in accordance with DSM-5 criteria. PSST for adolescents aged ≥18 years has excellent internal consistency, Cronbach's alpha of 0.91, and content validity of 0.91 [30].

### 2.4. Variables

Academic performance was the primary outcome variable. Self-reported cumulative grade point average (CGPA) was used as a proxy measure of academic performance. Premenstrual symptoms experience, history of depression, history of traumatic events, sleeping hour, pain during menses, length of menses, amount of menstrual bleeding, and duration of menses were the explanatory variables. Premenstrual symptoms experience rated as no/mild PMS, moderate/severe PMS, and severe PMS or PMDD. PMDD was diagnosed if at least one out of four “core PMS” symptoms rated as “severe,” at least four additional PMS symptoms rated either “moderate” or “severe,” and at least one out of five “area of functioning” items rated “severe.” PMDD was the secondary outcome variable. Moderate/severe PMS was diagnosed if at least one out of four “core PMS” symptoms rated either “moderate” or “severe,” at least four additional PMS symptoms rated either “moderate” or “severe,” and at least one out of five “area of functioning” items rated “moderate” or “severe” [31]. If the students did not fulfill the above classifications, they were diagnosed as “no/mild PMS.” Level of pain and amount of bleeding during menses were measured and rated based on the subjective experience of students.

## 3. Data Processing and Analysis

Data was coded, entered, and cleaned using EPI Info version 3.5.1. Before analysis, continuous variables were grouped based on previous research and internationally recommended cut-off value [1]. To examine the risk factors of PMDD, first, all variables were fitted to the bivariate logistic regression model. Then, variables were included in the final multiple logistic regression model if *p* value reached 0.05. Finally, independently associated risk factors were identified based on 0.05 significance level. The strength of association was determined using odds ratios with 95% confidence interval. Two-way ANOVA test of association was applied to investigate the association between academic performance and mental and reproductive health-related factors. In addition, Bonferroni post hoc test was done for multiple comparisons of groups difference in academic performance. Statistical Package for Social Science (SPSS) version 20.0 (IBM SPSS Corp.) was used for data analysis. The study was adherent to the strengthening of the reporting of observational studies in epidemiology (STROBE) statement (see Supplementary File 1: STROBE statement in the Supplementary material available online at https://doi.org/10.1155/2017/9348159).

## 4. Ethics Approval and Consent to Participate

Debre Berhan University, Institute of Health Science and Medicine Ethical Review Board, approved the study protocol. Participation was voluntary and data was collected anonymously after obtaining written consent from each student.

## 5. Results

### 5.1. Biopsychosocial Characteristics 

In total, 529 students completed the self-administered questionnaire with the response rate of 80%. The main reasons for nonresponse were a lack of interest and shortage of time. The mean age of students was 20.5 years (SD ± 1.54). More than two-thirds (70.1%) of students were single, 50.3% were first year, and 72.4% were from Amhara ethnic group. On average students slept 8 (SD ± 2.22) hours per day. The average menstrual cycle was 29.1 days (SD ± 9.5). More than half (54.3%) of students suffered from moderate pain during menses. The mean duration of menses was 4.31 days (SD ± 1.45). Furthermore, 13.2% of students reported heavy menstrual bleeding.

### 5.2. Premenstrual Symptoms Experience

**Figure 1:**
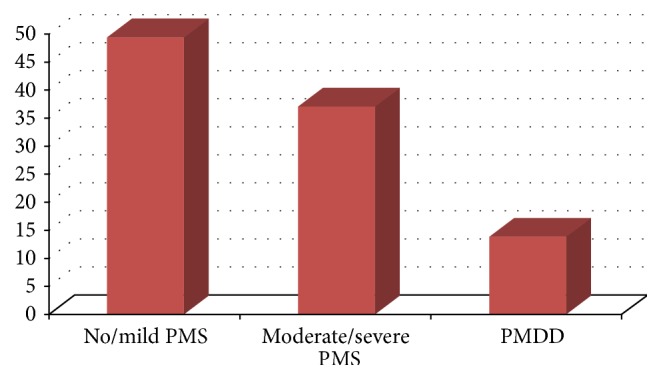
Frequency distribution of premenstrual symptoms experience, June 2015.

Almost all students (95.5%) had at least one mild premenstrual symptom, and 85.8% had moderate or severe symptoms. Fatigue or lack of energy (89.2%) was the most prevalent symptom followed by decreased interest in home activity (76.1%). Insomnia (50.1%) and overeating (46.7%) were the least prevalent symptoms (Table 1) Based on the DSM-5 criteria, 49.3% students were diagnosed with no/mild PMS, 36.9% moderate/severe PMS, and 13.8% severe PMS or PMDD (Figure 1).

## 6. Academic Performance

The mean cumulative grade point average (CGPA) was 2.66 (SD ± 0.51). Furthermore, 97.4% of students had the CGPA of ≥2.0 and 46.5% of students had above the mean CGPA. The ANOVA test of association revealed that the mean CGPA of students who fulfilled PMDD criteria was not significantly different from students without PMDD (*F-*statistic = 0.08, *p* value = 0.93). On the other hand, the mean CGPA of students with <21 days, 21–35 days, and >35 days of length of menses was significantly different. Multiple comparisons following Bonferroni post hoc test showed that the mean CGPA of students with <21 days of length of menses was significantly low compared to students with 21–35 days (*p* value = 0.023) and >35 days (*p* value = 0.006) of length of menses (Table 2).

## 7. Associated Factors of PMDD

The bivariate statistical modeling showed that pain during menses, amount of menstrual bleeding, maternal history of PMS, history of depression, psychiatric morbidity other than depression, and history of traumatic event were the significant risk factors of PMDD. Following multivariate modeling only pain during menses, amount of menstrual bleeding, and treatment seeking behavior persisted as a significant associated risk factor of PMDD.

Students who had severe pain during menses were 6.5 times more likely to develop PMDD compared to those who had no pain (AOR = 6.5; 95% CI = 1.6–43.7). Students who had heavy menstrual bleeding were 2.8 times more likely to develop PMDD compared to those who had minimal bleeding (AOR = 2.8; 95% CI = 1.2–7.1). Moreover, students who took the nonprescribed drug particularly pain-killers during menses were 8.5 times more likely to develop PMDD as compared to students who have used other options to manage symptoms (AOR = 8.5; 95% CI = 1.5–46.6) (Table 3).

## 8. Discussion

There was no significant association between premenstrual symptoms (PMS) experience and academic performance, but on the other hand, the length of menses significantly associated with academic performance. Based on the DSM-5 criteria, 49.3% of students were diagnosed with no/mild PMS, 36.9% moderate/severe PMS, and 13.8% severe PMS or PMDD. Pain during menses, amount of menstrual bleeding, and treatment seeking behavior were risk factors of premenstrual dysphoric disorder (PMDD).

The first objective of this study was to assess the association between academic performance and mental and reproductive health-related factors. In contrary to another study in Ethiopia [27], the current study showed that there was no significant difference in academic performance between students who fulfilled PMDD criteria and without PMDD. This does not imply that PMDD had no relevant effect on student academic performance. Therefore, this nonsignificant result might be due to two reasons. Primarily, this study had used cumulative grade point average (CGPA), which might be affected by previous semester or year grade. This justification was supported by the finding that half of the students were second year and above. Secondly, the PMDD was assessed based on one cycle of menstrual symptoms experience. This study found a significant association between length of menses and academic performance. Students with short length of menses had low academic performance. This might be due to frequent menstrual pain and mood change that can affect study time and alertness during regular class.

The second objective of this study was to investigate PMS experience and associated factors. In this study, the prevalence of PMDD was 13.8%. This finding was twice the prevalence rate of PMDD in Kuwait students [32], but on the other hand, it was lower than the Pakistani study report [33]. This discrepancy might be due to the difference in sample size, study population, and use of PMDD assessment tool. The Kuwait study [32] included only 110 students from a single department and the data was collected by daily record of severity of symptoms (DRSP) for two menstrual periods. In addition, the Pakistani study [33] has used Moos Menstrual Distress Questionnaire.

In agreement with the previous studies [19, 27, 34], this study revealed that 85.8% of the students had at least one moderate or severe symptom. However, cross-sectional studies conducted in Thailand [15], Japan [16], and Iran [35] depicted that relatively higher percentage of students had at least one moderate/severe premenstrual symptom. The prevalence rate of moderate/severe PMS in our study was 36.9%. This was higher than some other studies [10, 12, 33, 36, 37]. Furthermore, our study found that 17.0% of students reported impaired family relationship related to PMS experience.

Similar to earlier studies [17, 38, 39], this study uncovered that students who had severe pain during menses and heavy menstrual bleeding were more likely to develop PMDD compared to their counterparts. The possible explanation was that the pain and heavy menstrual bleeding increased the sensitivity to rejection by peers, anxiety, tension, and irritability. Students who took nonprescribed drug, particularly pain-killers for self-medication attempt to reduce the physical symptoms of PMS, during menses were more likely to develop PMDD compared to students who have used other options to treat premenstrual symptoms. The possible explanation could be that nonprescribed drugs may cause decreased serotonin, progesterone, and allopregnanolone level, which has been considered as the etiology of PMDD [40, 41].

Unlike previous studies [32, 35, 42–44], this study did not show a significant association between PMDD and history of depression, history of any psychiatric morbidity other than depression, history of traumatic event, sleeping hour, history of physical illness, increased educational status, physical exercise, marital status, age at menarche, and maternal history of PMS. This inconsistency may be due to difference in socioeconomic status and health seeking behavior of students.

This study recruited considerably large number of students from all fields of study. In addition, PMDD was ascertaining using a standardized validated scale [45]. Moreover, this study examined the temporal association of PMDD and academic performance. However, this study has certain limitations. First, premenstrual symptoms were not tracked prospectively by daily record of severity of problems (DRSP), which is the gold standard. To differentiate PMDD from other psychiatric morbidities, the American Psychiatric Association recommended PMDD must be confirmed by prospective daily symptom ratings for a minimum of two consecutive symptomatic cycles [4]. Second, due to the cross-sectional nature of the study, causal association cannot be assumed between PMDD and identified risk factors, and academic performance and length of menses as well. Third, this study used sensitive questions and self-reported CGPA, and diagnosis of PMDD was made based on self-reported symptoms that possibly added recall and social desirability bias. Lastly, this study was conducted in one institution which might limit external validity of the study results; however, this is possibly compensated by the inclusion of students from different ethnic groups.

## 9. Conclusions

Given premenstrual symptoms are a frequent source of concern to women [11], this study concluded that at least 1 out of 3 students suffered from moderate/severe PMS and 1 out of 10 students fulfilled DSM-5 criteria of PMDD; however, there was no difference in academic performance. Significant association was found between length of menses and academic performance. Severe pain during menses, heavy menstrual bleeding, and taking nonprescribed drug were risk factors of PMDD. To prevent complication such as suicidal ideation [46], student clinic at university should play a leading role to increase awareness and diagnose early and treat PMDD promptly. This study has implication to render further evidence for clinician and researcher. In addition, this study will provide relevant information on PMDD and academic performance at university for stakeholders including government and funding agencies.

## Supplementary Material

Supplementary File 1: STROBE statement containing the checklist of items that included in the current study.

## Figures and Tables

**Table 1:** Premenstrual symptoms and functional interference, June 2015.

	Not at all		Mild		Moderate		Severe	
	No	%	No	%	No	%	No	%
*List of symptoms*								
Anger/irritability	180	34.0%	160	30.2%	139	26.3%	50	9.5%
Anxiety/tension	153	28.9%	162	30.6%	156	29.5%	58	11.0%
Tearfulness/increased sensitivity to rejection	239	45.2%	159	30.1%	102	19.3%	29	5.5%
Depressed mood/hopelessness	184	34.8%	140	26.5%	148	28.0%	57	10.8%
Decreased interest in work activity	137	25.9%	138	26.1%	174	32.9%	80	15.1%
Decreased interest in home activity	126	23.9%	167	31.6%	163	30.9%	72	13.6%
Decreased interest in social activity	158	29.9%	155	29.4%	148	28.0%	67	12.7%
Difficulty concentrating	206	38.9%	150	28.4%	119	22.5%	54	10.2%
Fatigue/lack of energy	110	20.8%	143	27.0%	165	31.2%	111	21.0%
Overeating/food craving	282	53.3%	158	29.9%	65	12.3%	24	4.5%
Insomnia/difficulty of sleeping	264	49.9%	119	22.5%	101	19.1%	45	8.5%
Hypersomnia (need more sleep)	150	28.4%	139	26.3%	143	27.0%	97	18.3%
Feeling overwhelmed or out of control	228	43.1%	144	27.2%	105	19.8%	52	9.8%
Physical symptoms (e.g., muscle pain, bloating)	130	24.6%	105	19.8%	146	27.6%	148	28.0%

*Area of functioning*								
Symptoms interfered work efficiency or productivity	163	30.8%	153	28.9%	162	30.6%	51	9.6%
Symptoms interfered relationship with coworkers	178	33.6%	148	28.0%	144	27.2%	59	11.2%
Symptoms interfered relationship with family	180	34.0%	144	27.2%	115	21.7%	90	17.0%
Symptoms interfered social life activity	171	32.3%	150	28.4%	150	28.4%	58	11.0%
Symptoms interfered home responsibility	174	32.9%	164	31.0%	127	24.0%	64	12.1%

**Table 2:** Mental and reproductive health-related correlates of academic performance, June 2015.

Variables		*N* = 529	Mean CGPA	95% CI of mean CGPA	*F*-statistic	*p* value
Premenstrual symptoms						
No/mild PMS		261	2.67	2.61–2.73	0.08	0.93
Moderate/severe PMS		195	2.66	2.58–2.73		
PMDD		73	2.64	2.53–2.76		
History of depression						
Yes		48	2.69	2.54–2.85	0.19	0.66
No		481	2.65	2.61–2.70		
History of any psychiatric morbidity other than depression						
Yes		57	2.60	2.48–2.72	0.84	0.36
No		472	2.66	2.62–2.71		
History of traumatic event						
Yes		96	2.65	2.55–2.76	0.03	0.86
No		433	2.66	2.61–2.71		
Sleeping status						
≤5.9 hours/day		44	2.65	2.48–2.82	0.22	0.80
6.0–7.9 hours/day		104	2.63	2.54–2.73		
≥8.0 hours/day		380	2.67	2.62–2.72		
Pain during menses						
No		56	2.67	2.53–2.82	0.61	0.60
Minimal		118	2.71	2.61–2.80		
Moderate		257	2.65	2.58–2.71		
Severe		98	2.62	2.52–2.71		
Length of menses						
<21 days		21	2.36	2.18–2.54	5.15	0.006
21–35 days		487	2.66	2.62–2.71		
>35 days		21	2.85	2.61–3.09		
Amount of menstrual bleeding						
Minimal		103	2.61	2.51–2.71	1.06	0.35
Moderate		356	2.68	2.63–2.73		
Heavy		70	2.62	2.49–2.75		
Duration of menses						
≤3 days		166	2.64	2.56–2.71	0.39	0.53
≥4 days		363	2.67	2.61–2.72		

**Table 3:** Bivariate and multiple logistic regression model of biopsychosocial risk factors and PMDD, June 2015.

Variables (reference category)	PMDD status		Crude OR (95% CI)	Adjusted OR (95% CI)
	*n*/% (no)	*n*/% (yes)		
Current age, ≥20 (≤19)	356/67.3	58/10.9	1.1 (0.6–2.1)	
Marital status (single)				
Engaged	109/20.6	19/3.6	1.2 (0.6–2.1)	
Married	24/4.5	6/1.1	2.0 (0.6–4.1)	
Ethnicity (Amhara)				
Oromo	67/12.7	12/2.2	1.1 (0.5–2.1)	
Tigre	20/3.8	2/0.4	0.6 (0.1–2.2)	
Others	40/7.6	5/1.0	0.8 (0.3–1.8)	
Religion (orthodox)				
Muslim	22/4.2	2/0.4	0.5 (0.1–1.8)	
Protestant	51/9.6	5/1.0	0.6 (0.2–1.4)	
Field of study (natural and computational science)				
Agriculture science	55/10.3	9/1.7	1.2 (0.5–2.6)	
Social science	102/19.3	15/2.8	1.1 (0.5–2.1)	
Business and economics	75/14.2	21/4.0	2.0 (1.1–3.8)	
Health science	34/6.4	2/0.4	0.4 (0.1–1.5)	
Bach (first year)				
Second year	134/25.3	24/4.5	1.2 (0.7–2.1)	
Third year	90/17.0	15/2.8	1.1 (0.6–2.2)	
Daily sleeping hour (<5.9 hrs)				
6.0–7.9 hrs	90/17.0	14/2.6	0.7 (0.3–1.9)	
≥8 hrs	329/62.2	51/9.6	0.7 (0.3–1.7)	
Age at first menses ≥ 15 years (≤14 years)	296/56.1	50/9.4	1.2 (0.7–2.0)	
Pain during menses (no pain)				
Minimal	108/20.4	10/1.9	2.5 (0.6–16.6)	2.2 (0.5–15.5)
Moderate	228/43.1	29/5.5	3.4 (1.0–21.6)	2.8 (0.7–18.6)
Severe	66/12.5	32/6.0	13.1 (3.7–83.1)^*∗*^	6.5 (1.6–43.7)^*∗*^
Amount of menses (minimal)				
Moderate	318/6.1	38/7.2	1.0 (0.5–1.9)	1.1 (0.5–2.5)
Heavy/severe	47/8.9	23/4.3	3.7 (1.7–8.3)^*∗*^	2.8 (1.2–7.1)^*∗*^
Contraceptive use (nonuse)	23/4.3	3/0.6	0.8 (0.2–2.4)	
Length of menses (≤21 days)				
21–35 days	417/78.8	70/13.2	1.6 (0.5–10.1)	
≥35 days	20/3.8	1/0.2	0.5 (0.02–5.4)	
Menses for ≥4 days (≤3 days)	311/58.8	52/9.8	1.2 (0.7–2.0)	
Treatment seeking behavior (other)				
Nonprescribed drug	8/1.6	8/1.6	14.0 (3.0–64.5)^*∗*^	8.5 (1.5–46.6)^*∗*^
Prescribed drug	50/9.4	12/2.2	3.4 (0.9–12.7)	2.6 (0.6–10.8)
Sleep	252/47.6	35/6.6	2.0 (0.6–6.6)	1.7 (0.5–6.7)
Exercise	54/10.2	6/1.1	1.6 (0.4–6.6)	1.4 (0.3–6.8)
Diet change	50/9.4	9/1.7	2.5 (0.6–9.9)	2.0 (0.5–9.0)
History of depression (no)	36/6.8	12/2.2	2.3 (1.1–4.5)^*∗*^	1.5 (0.6–3.2)
History of psychiatric morbidity other than depression (no)	44/8.3	13/2.4	2.0 (1.1–3.9)^*∗*^	1.3 (0.6–2.8)
History of physical morbidity (no)	157/29.7	28/5.3	1.2 (0.7–1.9)	
Parental history of depression (no)	31/5.9	5/0.9	1.0 (0.3–2.5)	
History of traumatic event (no)	76/14.4	20/3.8	1.9 (1.1–3.3)^*∗*^	1.5 (0.8–2.8)
Mothers history of PMS	51/9.6	17/3.2	2.4 (1.3–4.4)^*∗*^	1.8 (0.8–3.5)
Physical exercise	80/15.1	16/3.0	1.3 (0.7–2.3)	

^*∗*^Statistically significant at *p* value 0.05.
